# A round robin approach to the analysis of bisphenol a (BPA) in human blood samples

**DOI:** 10.1186/1476-069X-13-25

**Published:** 2014-04-01

**Authors:** Laura N Vandenberg, Roy R Gerona, Kurunthachalam Kannan, Julia A Taylor, Richard B van Breemen, Carrie A Dickenson, Chunyang Liao, Yang Yuan, Retha R Newbold, Vasantha Padmanabhan, Frederick S vom Saal, Tracey J Woodruff

**Affiliations:** 1 Division of Environmental Health Sciences, University of Massachusetts – Amherst, School of Public Health, Amherst, MA, USA; 2 Department of Laboratory Medicine, University of California – San Francisco, San Francisco, CA, USA; 3 Wadsworth Center, New York State Department of Health, and State University of New York at Albany, Albany, NY, USA; 4 Division of Biological Sciences, University of Missouri, Columbia, MO, USA; 5 College of Pharmacy, University of Illinois, Chicago, IL, USA; 6 Program on Reproductive Health and the Environment, Department of Obstetrics, Gynecology, and Reproductive Sciences, University of California – San Francisco, San Francisco, CA, USA; 7 Wadsworth Center, NY State Department of Public Health, Albany, NY, USA; 8 National Institute of Environmental Health Sciences, Research Triangle Park, NC, USA; 9 Department of Pediatrics and Reproductive Sciences Program, University of Michigan, Ann Arbor, MI, USA

**Keywords:** Biomonitoring, Contamination, Endocrine disruptor, Linearity, Metabolite, Toxicokinetics

## Abstract

**Background:**

Human exposure to bisphenol A (BPA) is ubiquitous, yet there are concerns about whether BPA can be measured in human blood. This Round Robin was designed to address this concern through three goals: 1) to identify collection materials, reagents and detection apparatuses that do not contribute BPA to serum; 2) to identify sensitive and precise methods to accurately measure unconjugated BPA (uBPA) and BPA-glucuronide (BPA-G), a metabolite, in serum; and 3) to evaluate whether inadvertent hydrolysis of BPA-G occurs during sample handling and processing.

**Methods:**

Four laboratories participated in this Round Robin. Laboratories screened materials to identify BPA contamination in collection and analysis materials. Serum was spiked with concentrations of uBPA and/or BPA-G ranging from 0.09-19.5 (uBPA) and 0.5-32 (BPA-G) ng/mL. Additional samples were preserved unspiked as ‘environmental’ samples. Blinded samples were provided to laboratories that used LC/MSMS to simultaneously quantify uBPA and BPA-G. To determine whether inadvertent hydrolysis of BPA metabolites occurred, samples spiked with only BPA-G were analyzed for the presence of uBPA. Finally, three laboratories compared direct and indirect methods of quantifying BPA-G.

**Results:**

We identified collection materials and reagents that did not introduce BPA contamination. In the blinded spiked sample analysis, all laboratories were able to distinguish low from high values of uBPA and BPA-G, for the whole spiked sample range and for those samples spiked with the three lowest concentrations (0.5-3.1 ng/ml). By completion of the Round Robin, three laboratories had verified methods for the analysis of uBPA and two verified for the analysis of BPA-G (verification determined by: 4 of 5 samples within 20% of spiked concentrations). In the analysis of BPA-G only spiked samples, all laboratories reported BPA-G was the majority of BPA detected (92.2 – 100%). Finally, laboratories were more likely to be verified using direct methods than indirect ones using enzymatic hydrolysis.

**Conclusions:**

Sensitive and accurate methods for the direct quantification of uBPA and BPA-G were developed in multiple laboratories and can be used for the analysis of human serum samples. BPA contamination can be controlled during sample collection and inadvertent hydrolysis of BPA conjugates can be avoided during sample handling.

## Background

Human exposure to bisphenol A (BPA), a high production volume chemical, is ubiquitous due to its widespread use in numerous products including polycarbonate plastics and epoxy resins such as those used to line food and beverage containers [1,2], in medical equipment, thermal paper, and personal care products [3-8]. While the primary source of BPA exposure is through food, there is uncertainty with regard to the amount of exposure that can also occur dermally and through air [4,9-11].

Concerns surrounding BPA have been largely due to its estrogenic activity *in vitro* and *in vivo*[12]; BPA binds membrane estrogen receptor (mER), a transmembrane ER called G protein-coupled receptor 30 (GPR30), nuclear ERα and ERβ, and the orphan nuclear receptor estrogen related receptor-γ (ERRγ) [13-17]. Although it has been termed a weak estrogen, recent studies have shown that BPA produces non-genomic estrogen-like actions with the same potency and efficacy as estradiol [18-21]. In addition to its estrogenic properties, recent evidence from the US EPA and US NTP’s ToxCast program indicates that BPA interacts with a number of other receptors and pathways as well, including the androgen and thyroid signaling pathways [22].

A large number of rodent studies have shown that BPA can adversely affect endpoints including the development of the male and female reproductive tracts, obesity and other aspects of metabolism, development of the brain and neurobehaviors, and development of the mammary gland and its response to chemical carcinogens (reviewed in [23-27]). Importantly, many of these studies show effects from doses that are comparable to estimated human exposures (i.e. doses below 10 μg/kg/day [3,12,23,26-28]).

More than forty urine biomonitoring studies have shown that BPA metabolites are present in the vast majority (typically >90%) of individuals (reviewed in [10,29]). Specifically, studies of large reference populations from the United States, Canada, Germany and China demonstrate that BPA metabolites were measured in urine with central tendencies in the range of 1–3 ng/ml [30-33], although the upper percentiles of exposure often include individuals with concentrations in the 15–50 ng/ml range [30,34] or higher [35].

In recent years, there have been strong demands from the scientific community for measurements of circulating BPA in human blood, serum and/or plasma. There are several rationales for collecting these data. First, it has been suggested that making comparisons between administered doses that cause harm in animals and environmental exposures that may cause harm in humans requires accurate knowledge of circulating unconjugated BPA (uBPA) concentrations; BPA conjugates are not thought to bind to the estrogen receptor [36], although they may have other biological activities [37]. Thus, it has been argued if circulating concentrations of uBPA are not known in humans, it is not possible to say whether animal studies producing various concentrations of circulating uBPA are relevant. Second, given our current state of knowledge, measurements of BPA metabolites in urine cannot be used to predict serum unconjugated BPA (uBPA), since back-calculations to estimate serum uBPA require measurements of total BPA in a 24-hr urine sample as well as knowledge of all routes of exposure. BPA is rapidly metabolized when absorption occurs only from the GI tract after oral gavage [38,39]. It has been proposed that if metabolism is so extensive that there is very little serum uBPA, the risk will be low as the metabolites are not estrogenic [36,40-43].

In the past decade, more than three dozen studies have examined samples collected from pregnant women, non-pregnant adults, and fetal umbilical cords to evaluate the extent to which BPA may be present in blood or serum (reviewed in [11,29], see also [44-63]). Although a few studies used ELISA to measure BPA, the majority used analytical chemistry methods, and although most examined a limited number of human subjects compared to the studies examining urine, a few large studies have been published [32,64,65]. The majority of these studies have reported concentrations of uBPA in at least some human blood and serum samples in the low ng/ml range. In contrast to these findings, two studies with relatively high limits of detection (1.14 – 5 ng/ml) [41,66], a study of pooled human serum samples [46], and a storage validation study of blood bank samples performed by the CDC [67], have been unable to detect BPA in blood samples.

There continue to be disputes of the validity of measuring uBPA in human blood [11,68], with several studies challenging the analytical methods used, the possible contamination of reagents with BPA, as well as concerns regarding BPA leaching from materials used for sample collection, storage and processing [42,69-72]. Some studies have gone to extensive lengths to control for contamination from collection materials (see [73,74] for example), yet these concerns remain. It is also plausible that the extraction methods utilized to analyze blood/serum samples could be deconjugating BPA metabolites, therefore causing overestimations of human exposure to uBPA, although this possibility has not previously been systematically examined.

To address these issues, we first evaluated the potential for contamination from collection and analytic materials and identified standard collection materials, water and analytical apparatuses that are free from BPA contamination. Second, we performed a multi-laboratory round robin assay with participating laboratories receiving coded samples to determine the sensitivity, accuracy and precision of analytical chemistry methods (LC/MSMS) for the simultaneous detection of both uBPA and BPA-glucuronide (BPA-G), the major metabolite found in blood and urine in non-pregnant adults. We also used these direct methods to address the possibility that BPA-G was hydrolysed to uBPA during sample processing. Several participating laboratories compared these newly developed direct methods with indirect methods involving hydrolysis of conjugated BPA used in many previous studies, where unconjugated BPA and total BPA (BPA + BPA conjugates) are measured in separate assays. Finally, we applied these methods to determine the concentrations of uBPA and BPA-G in a small number of human serum samples.

## Methods

### Round robin design

We conducted the round robin in three phases, with a different set of samples collected for each phase. As discussed in more detail below, blood was collected and stored using materials that were determined to be free from BPA. All samples were sent to an NIEHS investigator for coding and redistribution, allowing for blinded analysis of samples in each laboratory. Data from each laboratory were returned to a single NIEHS investigator, who blinded the results so that they could be traced to a specific methodology but not a specific laboratory or investigator. A separate investigator, who was blinded to which laboratory produced which data, analysed the results. The round robin was conducted in the following three phases:

#### *Phase 1*

Two types of blank samples were collected and analysed by all participating laboratories: BPA-free water, and triple-stripped human serum (treated with dextran-coated charcoal three-times to remove steroids and BPA.) Pooled human serum was purchased (MP Biomedical), triple-stripped with charcoal [Sigma-Aldrich, St. Louis, MO], and spiked with one of five concentrations of uBPA (Sigma-Aldrich, purity >99%) and BPA-G (Sigma-Aldrich, purity 95%) (Table 1). An error in coding of these samples occurred; their results are not reported due to this error. A final set of serum samples was collected from human patients seeking care at San Francisco General Hospital with unknown health statuses (referred to as environmental samples) and pooled under two conditions: 1) using collection materials that had been shown to leach BPA (n = 5 pooled samples); or 2) using collection materials that were shown not to leach BPA (n = 5 pooled samples). All laboratories used their in-house standards for measurement of BPA.

**Table 1:** Spiked concentrations of BPA and BPA-glucuronide in three phases of round robin

**Phase #**	**Sample #**	**Sample type**				**Concentration of spiked uBPA (ng/ml)**	**Concentration of spiked BPA-G (ng/ml)**
		**Pooled human serum**	**Experimental blanks**	**Environmental sample**	**Commercial rat serum**		
1	1	X				0.2	2
	2	X				0.8	4
	3	X				3.2	8
	4	X				6.4	16
	5	X				12.8	32
	6	X				-	-
	7		water			-	-
	8		stripped serum			-	-
	9-13			X^a^		-	-
	14-18			X		-	-
2^b^	1	X				0.5	0.5
	2	X				1.3	1.3
	3	X				3.1	3.1
	4	X				7.8	7.8
	5	X				19.5	19.5
	6	X				-	-
	7-11			X		-	-
3^b^	1	X				0.5	0.5
	2	X				1.3	1.3
	3	X				3.1	3.1
	4	X				7.8	7.8
	5	X				19.5	19.5
	6	X				-	-
	7	X				-	3.1
	8	X				-	-
	9				X	0.09	0.8
	10				X	-	2.3
	11				X	-	-
	12-17			X		-	-

^a^Samples were collected with materials known to introduce BPA contamination. ^b^Non-stripped human serum used for spiked samples.

#### *Phase 2*

Serum was collected from multiple individuals, pooled, and spiked with one of five concentrations of uBPA and BPA-G (Table 1). These samples were not charcoal-stripped. An additional set of samples was collected from five individuals and left unspiked (environmental samples). For Phase 2 studies, the same authentic standards for BPA (Sigma-Aldrich, purity >99%) and D6-BPA (CDN Isotopes, Quebec, Canada, purity 98%) were used by all participating laboratories; authentic standards are highly characterized, highly pure compounds typically used as performance calibrators. While a standard for BPA-G was available when this phase began, isotopically labelled BPA-G was not available.

#### *Phase 3*

Serum was again collected from multiple individuals (healthy donors), pooled, and spiked with one of five concentrations of uBPA and/or BPA-G (Table 1). These samples were not charcoal stripped. Six additional samples were collected from four healthy human donors and left unspiked (environmental samples). All donors were provided with instructions for avoiding sources of BPA (including polycarbonate plastics, canned foods and dermal contact with thermal papers) during the 48 hr period prior to blood collection. A third set of three samples was generated by spiking commercially available rat serum; one sample contained no added BPA, one was spiked with BPA-G, and one was spiked with both uBPA and BPA-G. In Phase 3, authentic standards for BPA-G and ^13^C-BPA-G (produced by the National Toxicology Program) were used by all participating laboratories, along with the standards and isotopically labeled BPA, thus allowing for isotope-dilution assays to be conducted for both uBPA and BPA-G. One laboratory reported a sample handling error in this Phase and had to repeat the analysis. Due to this error, this laboratory (Laboratory #1) did not have sufficient sample remaining to analyse the rat serum.

### Laboratory participation

Four laboratories participated in the Round Robin experiments: UCSF (PI: Gerona), University of Illinois at Chicago (PI: van Breemen), Wadsworth Center (PI: Kannan), and University of Missouri at Columbia (PI: Taylor). Each laboratory had previous experience with the analysis of environmental chemicals in blood/serum samples, and several laboratories have published results on BPA in human, primate and rodent blood/serum samples [48,61,75]. Thus, for this validation experiment, each laboratory conducted analyses using their own equipment and methodologies. All four laboratories used methods that allowed uBPA and BPA-G to be measured simultaneously. Details about the analytical and detection methods used in each of these laboratories are provided in Tables 2 and 3 and in Additional file 1: Table S1.

**Table 2:** Extraction methods used in 4 participating laboratories

	**Laboratory #1**	**Laboratory #2**	**Laboratory #3**	**Laboratory #4**
**Volume used in analysis**	200 μL	500 μL	250 μL	1000 μL for spiked samples, 550 - 1000 μL for environmental samples
**Extraction protocol**	Protein precipitation used Honeywell Burdick & Jackson LC-MS grade acetonitirile containing 10 ng/mL [d6]-BPA.	Solid phase extraction: A Strata® NH_2_ cartridge (#8B-S009-FBJ; 200 mg/3 cc; Phenomenex, Torrance, CA) mounted on an Oasis® MCX cartridge (#186000254; 60 mg/3 cc; Waters, Milford, MA) was used.	Solid phase extraction: Waters Oasis HLB cartridge, 1 cc, 10 mg REF 18600383.	Solid phase extraction: Thermo Hypersep C18 (Thermo Fisher Scientific, cat # 60108–518), pre-washed with 15 ml methanol.
	Vortex mixed, centrifuged and removed 900 μL of supernatant; evaporated supernatant to dryness under stream of nitrogen.	Formic acid (98.2%; #F-4636) Ammonium acetate (98%; #0596-01), acetic acid (99.9%; #V194-04), hydrochloric acid (HCl, 37%; #H611), ammonium hydroxide (NH_4_OH, 29.5% assayed as NH_3_; #1177-04), and methanol (HPLC grade; #9093-03)	Honeywell B&J Methanol REF BJ230-4	HPLC-grade methanol (Fisher A452-4), water (Fisher W5-4) and ammonium acetate (**≥97%,** Fisher A639-500). Cartridges were washed with 25 mM ammonium acetate and water. Analytes were eluted with MeOH and dried under N2.
			Aqua Solutions Ultra Pure Water, HPLC grade, BPA free REF W1089-10 L	
	Reconstituted in 50 μL acetonitrile/water (50:50; v/v) (Honeywell Burdick & Jackson) and immediately analyzed using LC-MS-MS.		SPE column was washed with 5 column volumes methanol	
			Column activated with 1000 μL BPA free water	
			Load sample	
			Wash with 1000 μL 5% (v/v) methanol	
			Elute with 1000 μL pure methanol	
**Extraction pH**	not monitored	5.0	7 – 7.5	5.0
**Containers for extraction**	Fisher polypropylene micro-centrifuge tubes	16 × 100 mm Borosilicate Glass Disposable Culture Tube (#73500-16100, Kimble Chase).	VWR 16X100 mm Test Tubes REF 60825–425 Kimble and Chase 13x110 Conical Tubes REF 73785–5	Borosilicate glass tubes (Fisher Scientific cat#14-961-26).
**Drying agent for extraction**	Nitrogen	Nitrogen	Nitrogen	Nitrogen
**Water type & source**	Honeywell Burdick & Jackson LC-MS grade	Milli-Q water was purified by an ultrapure water system (Barnstead International, Dubuque, IA) and verified to be BPA free	BPA-free water (verified to be BPA free)	HPLC-grade water from Fisher Scientific. It has always tested BPA-free.
**Temperature for extraction**	RT	RT for extraction, 30°C for concentration with nitrogen	Ambient RT (20-25°C)	RT for extraction, 37°C for concentration with nitrogen

**Table 3:** Detection and analytical methods utilized in the four participating laboratories

	**Laboratory #1**	**Laboratory #2**	**Laboratory #3**	**Laboratory #4**
**Detection method**	UHPLC-MS/MS using a Shimadzu (Kyoto, Japan) Nexera UHPLC system and Shimadzu LCMS-8080 triple quadrupole tandem mass spectrometer	Agilent 1100 series HPLC (Agilent Technologies Inc.,) interfaced with an Applied Biosystems API 5500 electrospray triple-quadrupole mass spectrometer (ESI-MS/MS; Applied Biosystems).	LC-MS/MS (Agilent LC 1260- AB Sciex 5500 Triple Quadrupole)	LC-MS/MS using a Thermo Surveyor TSQ plus connected to an integrated Thermo-Accela LC system.
**Blanks**	Charcoal/dextran stripped human serum	Milli-Q water. Trace levels of free BPA were found in procedural blanks in some batches (0.40-0.46 for Phase 2, 0.19-0.28 for Phase 3).	Double-charcoal stripped human serum	HPLC-grade water (Fisher Scientific; cat# W5-4).
**Blanks: parallel?**	Yes	Yes	Yes	Yes
**Blank values subtracted from environmental samples?**	Yes	Yes	Not required – no contamination found in blanks	Yes
**LLOQ**	0.10 ng/ml (uBPA)	0.01 ng/ml (uBPA)	0.1 ng/ml (uBPA and BPA-G)	0.13 ng/ml (uBPA)
	0.01 ng/ml (BPA-G)	0.05 ng/ml (BPA-G)		0.06 ng/ml (BPA-G)
**ULOQ**	50 ng/mL		40 ng/ml (uBPA and BPA-G)	
**Calculations for determining LLOQ, ULOQ**	FDA Guidance for industry biomedical method validation	The LOD and LOQ were calculated as 3 times (3*S*) and 10 times (10*S*) of the standard deviations (*S*) of five replicate analyses, using the lowest calibration standard (0.01 ng/mL)	a signal that has a S/N of at least 10 and is the lowest calibrant that allows a linear regression coefficient of at least 0.95	The LOD and LOQ were calculated as 3 times (3*S*) and 10 times (10*S*) of the standard deviations (*S*) of three replicate analyses, using the lowest calibration standard. The means of three assays are given.
	http://www.fda.gov/downloads/Drugs/Guidances/ucm070107.pdf			
	Standard procedure. C1 pg. 6 of Guidance			
**LOD**	0.02 ng/ml (uBPA) 0.002 ng/ml (BPA-G)	0.003 ng/ml (uBPA) 0.02 ng/ml (BPA-G)	0.05 ng/ml (uBPA and BPA-G)	0.04 ng/ml (uBPA) 0.02 ng/ml (BPA-G)

### Sample collection

Blood was collected in phlebotomy laboratories at UCSF and University of Missouri and handled according to IRB protocols at those institutions. Collection was done via a 21 gauge straight needle, a 23 gauge vacutainer butterfly (BD REF 367342 or Greiner REF 450096 [Fisher Scientific, Suwanee, GA]) or a SafetyGlide needle (BD REF 305918 [Fisher Scientific]) following venous puncture. Blood was collected from the cubital vein into a venous blood collection tube (BD REF 366441 [Fisher Scientific]).

Within 30 minutes of the blood draw, samples were centrifuged, and serum was separated and transferred to cryovial tubes (Corning #430488 [Fisher Scientific]) or polypropylene centrifuge tubes (Corning # 4300791 [Fisher Scientific]). For studies involving spiked samples, serum was pooled, spiked with uBPA and/or BPA-G (see Methods below), and then frozen at -20°C. For the assessment of environmental levels of BPA in individual serum samples (environmental samples), the sample was directly frozen at -80°C. The effect of shipping conditions on BPA blood concentrations was evaluated, and it was found that storage of serum at -20°C or 4°C had no effect on uBPA concentrations whereas storage at room temperature or 37°C led to decreased concentrations of uBPA in a time-dependent manner (Additional file 2: Figure S1).

### Spiking of serum samples

In all three phases, pooled human serum samples were spiked with known concentrations of uBPA and BPA-G (Table 1). A non-spiked sample was retained from the same pool of serum for each round and analysed in parallel; we subtracted the values obtained for this non-spiked sample from each laboratory’s measured spiked sample, which allowed us to determine the accuracy of the method as a distinct issue from contamination during sample collection. Samples of commercially available rat serum (Bioreclamation LLC, Hicksville, NY; Lot# 187248) used in Phase 3 studies were spiked with uBPA and/or BPA-G, and an unspiked serum sample was retained and analysed.

### Sample preparation & instrumental analysis

In all laboratories, serum was thawed at room temperature and combined with an internal standard, as described above in “Round Robin Design”; note that different standards were available during different phases of the Round Robin. Each laboratory extracted the serum samples according to their pre-established protocols (Table 2). All participating laboratories used HPLC with tandem mass spectrometry (LC/MSMS) to identify and quantify uBPA and BPA-G. Each laboratory used different equipment (as noted in Table 3) that had been previously used to quantify environmental chemicals in human and/or animal serum samples. Details about chromatography and mass spectrometry parameters are available in Additional file 1: Table S1.

### Verification of BPA-free collection materials

In institutions where blood collections were performed (UCSF and University of Missouri), known blank reagents (i.e. stripped human serum) were run through the sample collection materials (needle, syringe, vacutainer butterfly, vacutainer tube, transfer pipet, cryovial tube), and then subjected to the sample extraction and LC-MSMS analyses. Only when the quantification of blanks identified undetectable levels of BPA were the method, reagents and materials considered to be verified for future experiments.

### Blanks testing

Through the various phases of the round robin, every laboratory routinely evaluated procedural (internal) blanks to ensure that new sources of BPA contamination were not being introduced from within the laboratory. These procedural blanks were laboratory specific (Table 3). In all laboratories, blank reagents were run through the sample extraction protocol (interacting with pipet tips, test tubes, cartridges, conical tubes, sample vials, water, methanol) *plus* the liquid chromatography (injection needle, injection port, capillaries, column, mobile phase solvents, mobile phase reservoir) and mass spectrometry procedures (injection valve, ion source, collision cell, quadrupole detector).

In addition, experimental blanks were coded and included in each phase (Table 1). These blanks included BPA-free HPLC grade water, stripped human serum and commercially available rat serum. We did not consider unstripped human samples to be blanks because of previous studies reporting uBPA in human serum [29]. All blanks were submitted as blind samples to the laboratories.

### Comparison of direct and indirect methods

The indirect method of analysing BPA is standard for urine, and has been used in multiple laboratories that have analysed human serum [46,56]. For indirect methods, uBPA is first measured, followed by enzymatic treatment of a split sample to deconjugate BPA-G and BPA-sulfate, and a second measurement of total BPA is taken. To compare the direct and indirect methods, three laboratories (Laboratories 2, 3 and 4) repeated the analysis of the samples collected in Phase 3 using indirect methods that utilized the same reagents, apparatuses, etc. and samples as those used in the direct measurement. The three participating laboratories used three different methods and enzymes for the indirect analysis (Table 4), with one method designed to replicate the CDC’s methodology (Laboratory 2), one designed to replicate the NCTR/FDA’s methodology (Laboratory 3), and a third method that included a higher concentration of enzyme (Laboratory 4).

**Table 4:** Characteristics of enzymatic treatments used in indirect measures

	**Laboratory # 2**	**Laboratory # 3**	**Laboratory # 4**
Method source	US CDC [46]	US FDA/NCTR [76]	Designed with high enzyme concentration
Enzyme source	*Helix pomatia*	Helix pomatia Type H-3	Helix pomatia Type H-1
Enzyme info	Sigma-Aldrich	Sigma-Aldrich	Sigma Aldrich, G 0751
Number of units used	291.4 U	2 U	1000 U
Volume of sample	0.5 ml	0.25 ml	1 ml
pH of reaction	5	5.5	5
Length of reaction	12 h	2 h	Overnight (~18 h)
Temperature of reaction	37°C	37°C	37°C

### Statistical analyses

Following collection of data, a participant who had not been involved in the chemical analyses was provided with coded data precluding knowledge of the individual laboratory from which the data were generated. The first objective was to evaluate whether the laboratories could distinguish high from low spiked samples and accurately determine BPA values. To assess linearity, we calculated the slopes, y-intercepts and R^2^ values for all five spiked samples included in Phase 2 and Phase 3 for both uBPA and BPA-G. We also assessed linearity only in the lower concentration range, calculating the slopes, y-intercepts and R^2^ values for just the lowest three spiked concentrations.

To assess accuracy, we compared the amount of uBPA and BPA-G that was spiked into each sample to the reported concentration from each laboratory. We agreed *a priori* upon an acceptable rate of error of 20% in the spiked to reported concentration to assess the accuracy of each laboratory’s method. We considered a laboratory’s method “verified” for each phase if they achieved an accurate reading (within 20% of the actual spiked amount) for 4 of 5 spiked samples. Each laboratory could participate in subsequent phases of the round robin whether or not their method was “verified” in a previous phase. Laboratories made small changes to their methods between phases (i.e. changes to the threshold smoothing values used to quantify peaks) but no major methodological alterations were reported.

For the comparison of direct and indirect methods, we corrected the concentrations of uBPA and BPA-G for MW using the calculation: [Total BPA] = [uBPA] + 0.566[BPA-G]. We then compared the measured MW-adjusted total BPA concentrations reported to the concentrations of spiked total BPA. When the direct and indirect methods were compared for non-spiked samples, we examined the measurements of MW-adjusted uBPA and BPA-G concentrations to determine which method was more likely to report a higher or lower concentration relative to the actual spiked concentration.

## Results

### Verification of BPA-free collection materials

**Figure 1:**
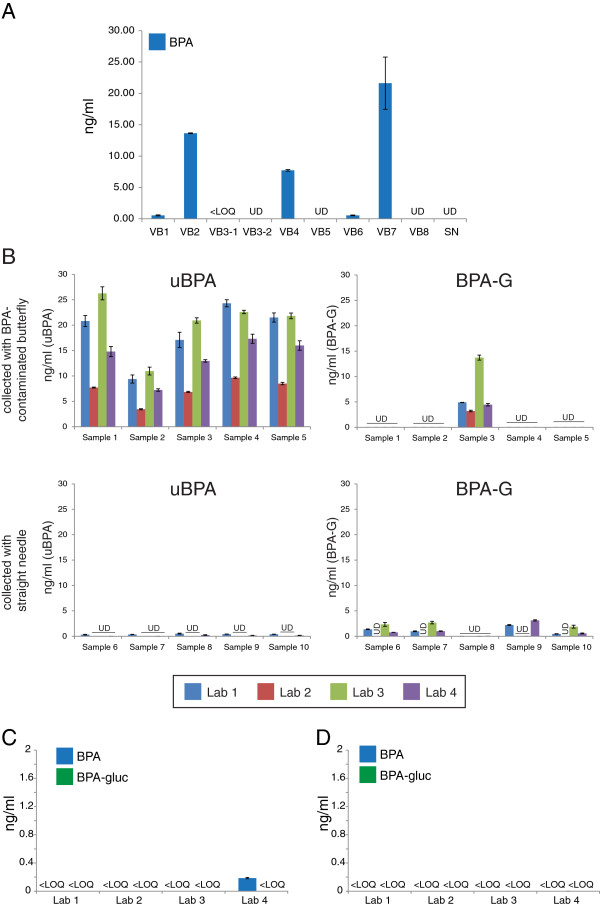
**Identification of collection materials free from BPA contamination. A)** Eight vacutainer butterflies (VB) and one straight needle (SN) were tested for BPA contamination. Double-stripped serum was run through these materials and tested by one participating laboratory. Vacutainer butterfly #3 (VB3) was tested twice. **B)** Samples collected with BPA-contaminated vacutainer butterflies were easily distinguished from samples collected with a straight needle. Very high uBPA concentrations were reported for all samples collected via contaminated collection materials by all laboratories whereas multiple samples collected with the straight needle had uBPA concentrations reported as undetectable (UD) from several laboratories. **C)** uBPA and BPA-G concentrations measured in blank water samples. Three laboratories reported no uBPA or BPA-G in any sample. Laboratory 4 reported a low concentration (0.17 ng/ml) of uBPA. **D)** uBPA and BPA-G were not reported at quantifiable levels in stripped human serum by any participating laboratory. In all panels, UD indicates undetected levels; <LOQ indicates detectable levels that were below the limit of quantification.

Each laboratory independently analysed their sample extraction protocol, processing materials (including pipet tips, test tubes, cartridges, conical tubes, sample vials, water, methanol) and their liquid chromatography (injection needle, injection port, capillaries, column, mobile phase solvents, mobile phase reservoir) and mass spectrometry procedures (injection valve, ion source, collision cell, quadrupole detector) to ensure that their materials and reagents did not introduce BPA contaminations in the laboratory (Table 3). In addition, to specifically assess collection materials, in one of the blood collection laboratories, BPA was detected in stripped human serum after processing through some vacutainer butterfly needles but not through other vacutainer butterfly needles or straight needles (Figure 1A). To evaluate the laboratories’ ability to detect contaminated serum samples, we collected blood samples under two different scenarios: 1) samples were collected via a vacutainer butterfly needle identified as contributing BPA during blood collection; and 2) samples were collected via a straight needle that was identified to be BPA free. uBPA concentrations were very high in samples collected with the vacutainer butterfly needle (>7 ng/ml), whereas concentrations measured in the samples collected via the straight needle ranged from < limit of detection (LOD) - 0.53 ng/ml (Figure 1B). In subsequent testing of the collection materials selected for the remainder of the Round Robin experiments, BPA was not observed in either water or stripped human serum, with the exception of a low concentration (0.17 ng/ml) measured in the water sample in one laboratory (Figure 1C,D).

### Linearity of uBPA and BPA-G in spiked serum samples

**Figure 2:**
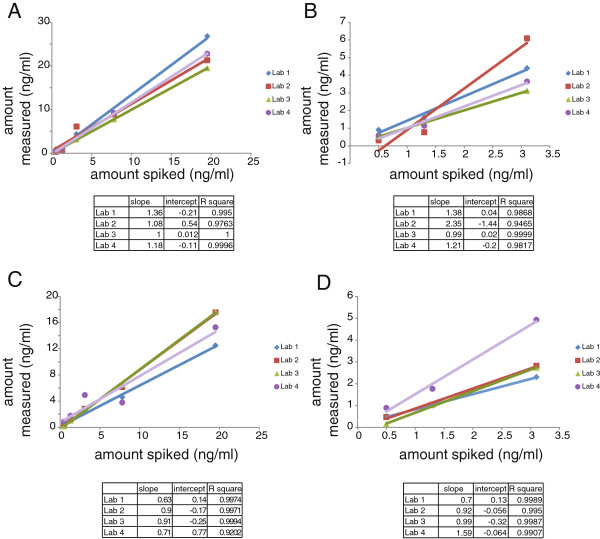
**Linearity was observed by all laboratories for uBPA and BPA-G in spiked serum samples. A)** Linear relationships were examined for uBPA in Phase 2 samples (spiked over the range of 0.5 to 19.5 ng/ml) by all four laboratories. **D)** Linearity analyses were limited to only the three samples spiked with the lowest concentrations of uBPA (0.5 to 3.1 ng/ml). All laboratories were still able to distinguish low, moderate and high concentrations of uBPA. **C)** Linear relationships were also examined for BPA-G in all Phase 2 samples (spiked over the range of 0.5 to 19.5 ng/ml) by all four laboratories. **B)** Linearity analyses were limited to only the three samples spiked with the lowest concentrations of BPA-G (0.5 to 3.1 ng/ml). All laboratories were still able to distinguish low, moderate and high concentrations.

In Phase 2, the slope of the relationship between spiked and measured samples ranged from 1.0 to 1.36 among the four laboratories for uBPA (R^2^ values 0.98 to 1.0) and 0.63 to 0.91 for BPA-G (R^2^ values 0.92 to 1.0) (Figure 2A,C). We observed similar results when the analysis was limited to the samples spiked with the three lowest concentrations, with slightly wider variation in the slope values but R^2^ values were essentially around 1 (Figure 2B,D). For Phase 3, slopes ranged from 0.89 to 1.08 for uBPA and 0.75 to 1 for BPA-G and R^2^ values were close to 1 over the entire range of doses examined, as well as over the three lowest concentrations (Additional file 3: Figure S2).

### Accuracy of analytical methods: results from spiked serum samples

**Figure 3:**
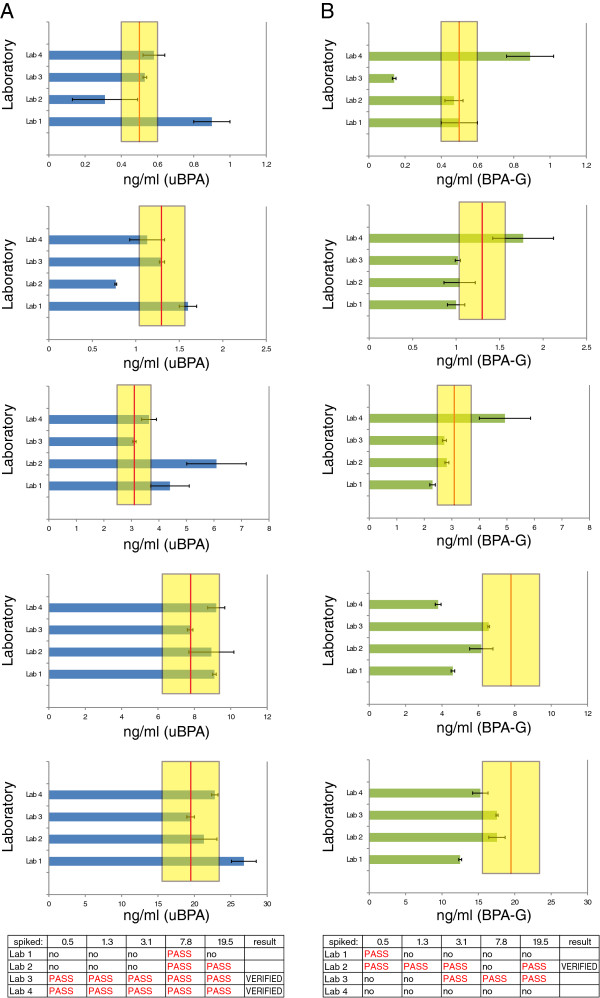
**Accuracy of uBPA and BPA-G measurements in five spiked samples from Phase 2. A)** Results reported for uBPA measurements in spiked samples by four participating laboratories. Each graph (top to bottom) represents the data from an individual spiked sample ranging from the lowest concentration (0.5 ng/ml) to the highest concentration (19.5 ng/ml). **B)** Results reported for BPA-G measurements in spiked samples by four participating laboratories. Each graph (top to bottom) represents the data from an individual spiked sample ranging from the lowest concentration (0.5 ng/ml) to the highest concentration (19.5 ng/ml). In both panels, graphs represent mean ± standard deviations reported from each laboratory. The red line marks the actual concentration spiked and the yellow bar marks the range of ±20%. At the bottom of each panel is the performance summary for each laboratory for Phase 2 for uBPA **(A)** and BPA-G **(B)**. A method was considered “verified” for the phase when at least 4 of 5 spiked samples measured concentrations within 20% of the actual spiked amount.

In both Phase 2 and Phase 3, uBPA and BPA-G were detected at low concentrations in the unspiked pooled samples (Additional file 4: Figure S3A, 3B). These concentrations were subtracted from the values reported from each laboratory for the spiked samples. For the five spiked samples, two laboratories had verified methods for the detection of uBPA and one laboratory had verified methods for the detection of BPA-G in Phase 2 (Figure 3). Laboratories that did not have validated methods in this Phase typically underestimated the concentrations of BPA-G, but concentrations of uBPA were both underestimated and overestimated. In Phase 3, three laboratories had verified methods for the detection of uBPA and two laboratories had verified methods for the detection of BPA-G (Additional file 5: Figure S4). Laboratory #1 typically overestimated concentrations of uBPA and BPA-G, whereas Laboratory #4, which was not validated for BPA-G, typically underestimated concentrations of this compound.

### Analysis of deconjugation of BPA-G during sample handling and analysis

**Figure 4:**
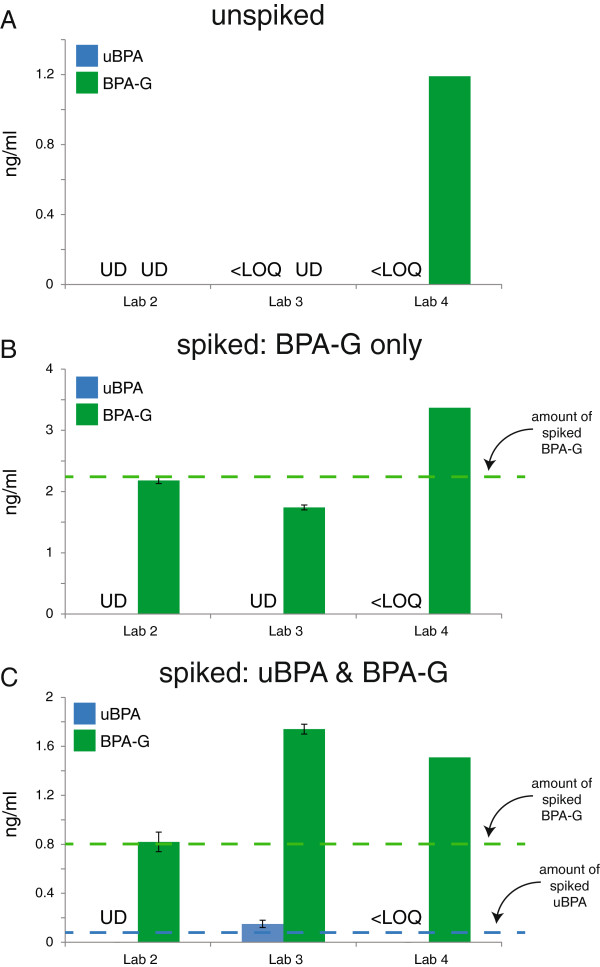
**Analysis of inadvertent hydrolysis of BPA-G in spiked rat serum. A)** Unspiked samples were analyzed for uBPA (blue) and BPA-G (green). uBPA was not detected by any laboratory; one of three laboratories reported BPA-G in this sample. **B)** Serum samples spiked with only BPA-G were analyzed for uBPA and BPA-G. uBPA was not measured above the LOQ by any laboratory. **C)** Sample spiked with uBPA concentrations at or near the LOD for the methods used by the participating laboratories (see Table 3), plus BPA-G. All three laboratories reported BPA-G, but only one measured uBPA at concentrations above the LOQ. For all panels, dotted lines indicate the concentrations spiked and graphs represent mean ± standard deviations reported from each laboratory with the exception of Laboratory 4, which could not perform replicate measures due to the volume of serum required for their assay and the limited amount of rodent serum available. UD indicates undetectable levels; <LOQ indicates detectable levels that were below the limit of quantification.

To determine whether deconjugation was occurring during sample handling and analysis, we analysed pooled human samples spiked only with BPA-G. All four laboratories reported that the majority of BPA detected in the spiked sample (92.2 – 100%) was in the form of BPA-G (Additional file 6: Figure S5); low concentrations of uBPA (<0.3 ng/ml) were detected by two of four laboratories. Because unstripped human serum is never considered ‘blank’, we repeated this experiment using commercially available serum from rodents that were not exposed to BPA. In the unspiked rat sample, uBPA was not detected by any of the three participating laboratories; BPA-G was detected by one laboratory (1.19 ng/ml) (Figure 4A). In the sample spiked with only BPA-G, all three laboratories reported measurable concentrations of BPA-G, and none reported uBPA (Figure 4B). A third sample was spiked with uBPA at or near the LOD and with a higher concentration of BPA-G. Only one of three laboratories detected uBPA; all three laboratories detected BPA-G, with two of three laboratories reporting concentrations higher than what was spiked (Figure 4C).

### Comparison of direct and indirect methods

**Figure 5:**
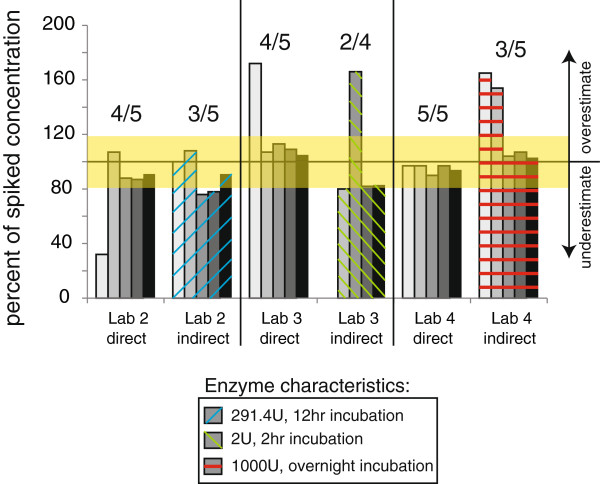
**Comparison of indirect and direct methods.** Three laboratories analyzed the phase 3 samples for total BPA (uBPA + BPA-G) using both direct and indirect methods. For the indirect methods, enzyme was used at the concentrations and duration of treatment indicated (see also Table 4). The indirect method used by Laboratory 2 replicates the protocol used by the CDC and the indirect method used by Laboratory 3 replicates the protocol used by NCTR/FDA. All three laboratories had verified methods for the analysis of Phase 3 samples, with at least 4 of 5 spiked samples concentrations reported within 20% of the actual spiked amount (indicated by the number over the suite of 5 samples). None of the indirect methods were verified using these criteria. The yellow bar shows 20% accuracy around the actual spiked amount.

Using indirect methods, Laboratory 2 reported three of the five samples had BPA levels within 20% of the spiked concentration compared to four out of five using the direct method (Additional file 5: Figure S4). The indirect method used by Laboratory 2, which was selected to replicate the methods used by the US CDC [46] typically underestimated the total BPA spiked. Similarly, both Laboratories 3 and 4 reported fewer BPA values within 20% of the spiked levels for the indirect versus the direct method (Figure 5). Results of the indirect method used by Laboratory 3, selected to replicate the methods utilized by the US FDA/NCTR in the analysis of BPA in blood [76], reported that only two of four spiked samples were within 20% of the total BPA concentration spiked compared to four of five spiked samples analysed with the direct method. Finally, the indirect method used by Laboratory 4, which used a higher concentration of enzyme compared to the CDC or FDA/NCTR methods (1000 U), reported three of five samples within 20% of the actual spiked concentration compared to five of five samples analysed with the direct method *(*Figure 5). Because the threshold for a verified method was four of five spiked samples within 20% of the actual spiked concentrations, all three direct methods were considered verified for total BPA in this phase; no indirect method was verified.

### Analysis of environmental samples reveals low levels of uBPA and BPA-G

**Figure 6:**
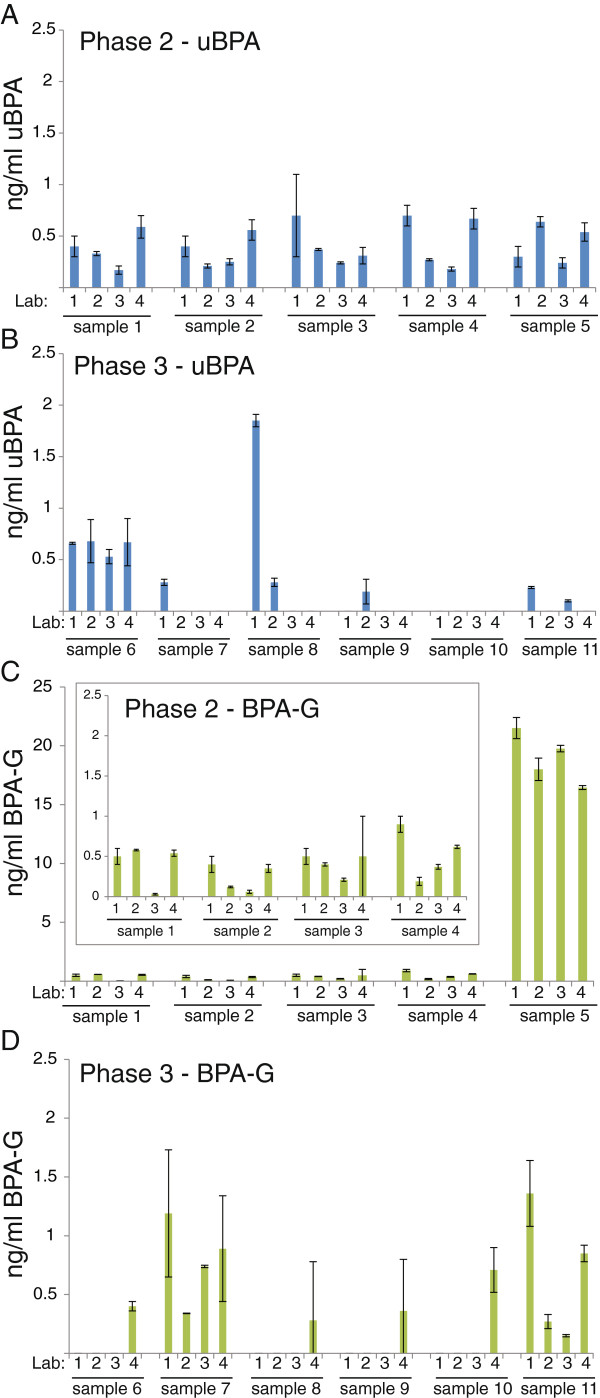
**Analysis of uBPA and BPA-G in environmental samples. **uBPA concentrations were analyzed in five environmental serum samples in Phase 2 **(A)** and six environmental serum samples in Phase 3 **(B)**. BPA-G concentrations were analyzed for the same five environmental serum samples in Phase 2 **(C)** and six environmental serum samples in Phase 3 **(D)**. Sample 5 in Phase 2 had high concentrations of BPA-G reported by all four laboratories, requiring this data to be presented with a different scale. See inset for better resolution of Samples 1–4. In all panels, graphs represent mean ± standard deviations reported from each laboratory.

In Phase 2, uBPA was detected in all five environmental samples by the laboratories at low concentrations (typically below 0.5 ng/ml) (Figure 6A). BPA-G was also detected in these samples, although one sample (Sample 5) had much higher concentrations reported by all laboratories compared to the other samples analysed [mean = 18.9 ng/ml, 36–81 times higher than the other four samples from this phase], (Figure 6C). In contrast, in Phase 3, where donors were instructed to avoid known BPA sources (canned foods, thermal papers, etc.), both uBPA and BPA-G were detected in fewer samples (Figure 6B,D).

## Discussion

This Round Robin was designed to address concerns that have been raised in the study of human exposures to BPA, focusing on three goals: 1) to identify collection materials, analytical reagents and detection apparatuses that do not contribute BPA to blood or serum samples; 2) to identify methods that can accurately measure uBPA and BPA-G in human serum samples and evaluate the performance of each individual laboratory; and 3) to evaluate whether inadvertent hydrolysis of BPA-G occurs during sample handling or processing.

To address the first goal, we tested numerous blood collection materials including vacutainer butterflies and straight needles to identify materials that could be used without introducing BPA contaminations. Other studies have reported that numerous collection materials, analytical reagents, and storage containers contain and/or leach BPA, but that these contaminations can be eliminated via careful screening and selection of materials and reagents [56,72,76,77]. Similar to the data reported in these studies, we identified blood collection materials contaminated with BPA, as well as contaminant-free collection materials, analytical reagents and detection apparatuses, and in almost every case the laboratories did not find BPA above the limit of quantification in BPA-free water or stripped human serum (Figure 1). Once blood collection materials were identified that did not leach or contribute BPA to charcoal dextran-stripped human serum, these verified materials and reagents were used throughout the remainder of the Round Robin experiments. Therefore, although extensive validations of dozens of materials were required, we found, similar to prior published reports, that external contaminations could be managed, allowing for the analysis of uBPA and BPA-G in human blood samples.

To address the second goal, to identify methods that can accurately measure uBPA and BPA-G in human serum samples, four laboratories analyzed more than 10 samples spiked with known concentrations of uBPA and BPA-G and assessed the accuracy of their measurements; all laboratories were blind to sample concentrations. Several laboratories met the reliability criteria established *a priori* (i.e. four of five samples in each phase with reported concentrations within 20% of the actual spiked amount), indicating that these laboratories can accurately quantify uBPA and/or BPA-G in human serum (Figure 3, Additional file 5: Figure S4). Additionally, all laboratories were able to distinguish between low, moderate and high concentrations of uBPA and BPA-G with R^2^ values above 0.9, indicating a high degree of linearity for both (Figure 2, Additional file 3: Figure S2).

As for the performance of each participating laboratory, for several laboratories, there was improvement between Phases 2 and 3 of the Round Robin (for uBPA: Laboratory 2 was verified in Phase 3, but not Phase 2; for BPA-G, Laboratory 3 was verified in Phase 3, but not Phase 2). These improvements may be due to the use of different standards between these phases, suggesting that the use of authentic standards such as D6-BPA and ^13^C-BPA-G may improve performance of laboratories that wish to quantify uBPA and BPA-G in human serum samples. Each laboratory used slightly different methods, which included different extraction, detection and analytical methods. From the design of this Round Robin, we are not able to determine which of these factors is responsible for the better performance of some laboratories than others. Yet, one implication of these results is the need for future studies to include quality control measures, including the use of spiked samples, to validate their individual methods.

Importantly, the four laboratories participating in this Round Robin used methods that allowed for simultaneous detection of uBPA and BPA-G; this is in contrast to prior methods that required two separate measures: the detection of uBPA, followed by the treatment of sample with enzyme to hydrolyze BPA conjugates and a second detection of total BPA (uBPA + BPA-G and BPA-sulfate). In all three laboratories that tested both indirect and direct methods, the direct methods were considered verified whereas the indirect methods were not (Figure 5). Specifically, enzyme treatment protocols that were designed to replicate the protocols used by the US CDC ([46] methods used by Laboratory 2) and the US FDA/NCTR in the analysis of BPA in blood ([76] methods used by Laboratory 3) tended to underestimate the concentrations of total BPA in spiked samples. The laboratory that used a higher concentration of enzyme (1000 U, Laboratory #4) overestimated total BPA levels in some samples using their indirect method. A limitation of the indirect method is that there is variability in the activity of the enzyme purchased for use in these assays, so the amount of enzyme used and the incubation time required to optimize the assay have to be determined for each batch of enzyme.

For the third goal, to evaluate whether inadvertent hydrolysis of BPA-G occurs during sample handling or processing, human and rodent serum samples were spiked with BPA-G and then analyzed to determine whether this conjugate was hydrolyzed during sample processing and handling. In all participating laboratories, only BPA-G was detected in spiked rodent serum samples (Figure 4); the lack of quantifiable levels of BPA indicates that these methods do not inadvertently deconjugate BPA-G. Similar results were obtained with the human samples, but these results are more difficult to interpret because the spiked samples were prepared with unstripped human serum which contained measurable levels of uBPA and BPA-G prior to spiking (Additional file 6: Figure S5).We also analyzed the concentrations of uBPA and BPA-G in a small number of environmental samples, collected from individuals with no interventions (Phase 2) or individuals that were instructed to avoid known sources of BPA (Phase 3). Using the contaminant-free collection and storage materials we identified, uBPA was detected in measurable quantities in some environmental samples, typically at concentrations below 0.5 ng/ml (Figure 6). Importantly, although uBPA concentrations measured in environmental samples were typically low or below the limits of detection, we observed high concentrations of total BPA in one individual (Phase 2, sample 5), who had BPA-G concentrations >16 ng/ml. This finding suggests that there may be individuals in the general population with high overall burdens of BPA, indicating that the range of BPA exposures may be larger than previously suggested. Because the high concentrations were observed for BPA-G, a biological metabolite not found in consumer products, inadvertent contamination is not possible.

More than forty published studies have reported low concentrations of BPA in human blood and serum samples (reviewed in [29], see also [44-63]). In spite of the relative consistency in BPA concentrations reported, these studies have been criticized that the uBPA measured in human serum could be the result of contamination and/or that BPA-G, the major metabolite found in blood, could be deconjugated by the extraction processes used [42,69-72]. Our results are not able to determine the validity of any previous findings on BPA in human serum, and cannot assess which of the published studies reporting BPA in human blood and serum might be affected by BPA contamination from collection materials, analytical reagents, storage containers, or the detection apparatuses used. Although many of these studies have reported information on the quality control measures undertaken to limit BPA contamination, others lack this information. Importantly, this Round Robin, like other studies [56,72,76,77], indicates that BPA contaminations can be controlled, and our analyses of environmental samples indicate that low concentrations of uBPA and BPA-G in human serum are plausible.

One reason why biomonitoring studies have been challenged is that toxicokinetic studies, in which known quantities of BPA are administered under controlled circumstances, suggest that very large oral doses are required to produce circulating blood levels of uBPA above the limits of detection of current methodologies [41,78]. Measures of BPA in consumer products [2,9,79] have been used to estimate daily human exposures of less than 5 μg/kg/day. Additionally, because daily output in urine is considered a good measure of 24-hour exposures, back-calculations from the concentrations of BPA measured in urine also estimate that daily exposures are less than 5 μg/kg/day [80-82]. When these low exposure estimates are combined with data from human oral toxicokinetic studies [41,78], models suggest that BPA should not be detected in human serum because expected circulating concentrations would be below the limits of detection [83]. There is a difference in the numerous studies reporting uBPA in human serum samples - including some of the environmental samples collected for this Round Robin – and expected blood concentrations calculated from toxicokinetic models, and there are several factors that can contribute to this difference. First, it is important to note that the human toxicokinetic studies conducted for BPA to date have limitations that can affect their accuracy. These include the use of analytical techniques with low sensitivity and high limits of detection (LOD = 1.14 ng/ml in [41] and LOD = 2.28 ng/ml in [78]) and the examination of a very small number of adults (n = 6 or 8 in [78], n = 6 in [41]) without taking into account how age, gender and other physiological factors can influence chemical metabolism [68]. Furthermore, these studies examined the disposition of BPA following acute oral exposures, including exposures via gelatin capsules, whereas actual human exposures occur via multiple exposure routes and are chronic, factors that will likely influence toxicokinetics [9,10,84,85]. Finally, a small number of animal studies have examined the disposition of BPA to tissues following exposure [86], but the possibility that BPA could bioaccumulate has not been well addressed [85].

Studies indicate that metabolism of BPA is dependent on route of exposure, and non-oral exposures have been shown to produce higher concentrations of circulating uBPA than exposures that occur via gavage [39,87,88]. For example, one recent study in canines showed that BPA absorption via the oral mucosa resulted in serum uBPA 100-fold greater both in terms of the percent bioavailable and average uBPA serum levels (based on area under the curve) compared to experiments where BPA was placed directly in the gut (via gavage) [39].

Ultimately, the results of this Round Robin cannot solve the dispute between toxicokinetic models predicting undetectable levels of uBPA in human blood and biomonitoring studies reporting measurable levels of uBPA (in the low or sub ng/ml range). To address this argument, a large number of variables need to be identified including all sources of human exposure, their relative contributions to total daily exposures, the timing of exposures throughout the day and between days, and replication of the toxicokinetic parameters that have been derived from limited studies; to date, no toxicokinetic study has replicated the repeated daily exposures via multiple routes experienced by humans. Nevertheless, this round robin study provides information pertaining to the need for exercising adequate caution during sampling and analysis of biospecimens for BPA. Furthermore, this study provides evidence that analysis of uBPA and BPA-G can be performed accurately at concentrations that are relevant to humans.

Ongoing conversations in the field of Environmental Health have debated whether future BPA studies should characterize exposures from urine or blood/serum. Urine has long been the preferred matrix for assessing human exposures to environmental chemicals because it is easy to obtain and can be collected without pain, an especially important consideration when studies include infants or children [89,90]. However, in cases where toxicokinetic parameters are calculated, analyses based solely on concentrations in urine will have significant uncertainties; urine concentrations can provide a snapshot of prior exposures, however they cannot be used to calculate blood concentrations of uBPA unless all of the sources and routes of exposure are known, as these factors significantly influence toxicokinetic parameters [84,91]. Thus, toxicokinetic studies require analysis of BPA in blood. In studies that use blood or serum, investigators need to report the details regarding steps taken to ensure the lack of contamination and should identify that they screened their collection materials, analytical reagents and storage materials to ensure that contamination was not introduced. Field blanks should also be assessed using appropriate matrices (i.e. charcoal dextran-stripped human serum).

## Conclusions

This Round Robin process identified LC/MSMS protocols in different laboratories that can be used to accurately measure uBPA and/or BPA-G in human serum. When these direct methods were applied to a small number of environmental samples, uBPA and BPA-G were detected in some but not all samples, typically at concentrations below 0.5 ng/ml. Future studies using these methods and larger numbers of samples collected with materials that have been verified to be contaminant free are needed to make conclusions about the frequency of detection and average concentrations in specific populations. Finally, toxicokinetic studies employing multiple exposures and different routes, reflecting real-world exposure scenarios, are needed to identify and evaluate the multiplicity of sources and routes of exposure experienced by the human population that may influence levels measured in human serum.

## Abbreviations

BPA: Bisphenol A; BPA-G: BPA-glucuronide; CDC: Centers for disease control and prevention (US); ELISA: Enzyme-linked immunosorbent assay; ERRγ: Estrogen related receptor γ; FDA: Food and Drug Administration (US); GPR30: G protein-coupled receptor 30; HPLC: High performance liquid chromatography; LOD: Limit of detection; LOQ: Limit of quantification; mER: Membrane estrogen receptor; MS/MS: Tandem mass spectrometry; MW: Molecular weight; NCTR: National Center for Toxicological Research; NIEHS: National Institute of Environmental Health Sciences; RT: Room temperature [this abbreviation is only used in tables]; uBPA: unconjugated BPA; UCSF: University of California – San Francisco.

## Competing interests

LNV provided expert testimony in a civil case involving a product that might contain EDCs. FSvS wrote a report for attorneys involved in product labeling litigation. RRG, KK, JAT, RBvB, CAD, CL, YY, RRN, VP, and TJW have no conflicts to disclose.

## Authors’ contributions

LNV, RRG, KK, JAT, RBvB, CAD, RRN, VP, FSvS and TJW designed the experiments. RRG, JAT, CAD, FSvS and TJW collected the blood samples. RRG, KK, JAT, RBvB, CL and YY analyzed the blood samples. RRN coordinated the coding and shipping of samples and coded the data. LNV, RRG, KK, JAT, RBvB, CAD, CL, YY, RRN, VP, FSvS and TJW analyzed the data. LNV and TJW wrote the paper and made the figures. LNV, RRG, KK, JAT, RBvB, CAD, CL, YY, RRN, VP, FSvS and TJW critically edited the paper. All authors read and approved the final manuscript.

## Supplementary Material

Additional file 1: Table S1 Liquid chromatography and mass spectrometry parameters used by Round Robin laboratories. Click here for file

Additional file 2: Figure S1 Analyses of shipping conditions. Serum samples were spiked with 500 ng/ml uBPA and subjected to different storage conditions for up to 7 days. uBPA concentrations were stable when stored at -20°C or 4°C, but unstable at room temperature or 37°C. Click here for file

Additional file 3: Figure S2 Linearity was observed by all laboratories for uBPA and BPA-G in spiked serum samples from Phase 3. A) Linear relationships were observed for uBPA in Phase 3 samples (spiked over the range of 0.5 to 19.53 ng/ml) by all four laboratories. B) When analyses were limited to only the three samples spiked with the lowest concentrations of uBPA (0.5 to 3.13 ng/ml), laboratories were still able to distinguish low, moderate and high concentrations of uBPA. C) Linear relationships were also observed for BPA-G in all Phase 2 samples (spiked over the range of 0.5 to 19.53 ng/ml) by all four laboratories. D) When analyses were limited to only the three samples spiked with the lowest concentrations of BPA-G (0.5 to 3.13 ng/ml), all laboratories were still able to distinguish low, moderate and high concentrations. Click here for file

Additional file 4: Figure S3 uBPA and BPA-G were detected in unspiked pooled samples that were used for different Round Robin experiments. A) Concentrations of uBPA and BPA-G reported for the Phase 2 pooled samples that were used for spiked experiments with uBPA and BPA-G. B) Concentrations of uBPA and BPA-G reported for the Phase 3 pooled samples that were used for spiked experiments with uBPA and BPA-G. C) Concentrations of uBPA and BPA-G reported for the Phase 3 pooled samples that were spiked with BPA-G only. In all panels, graphs represent mean ± standard deviations reported from each laboratory. Click here for file

Additional file 5: Figure S4 Accuracy of spiked samples, Phase 3. A) Results reported for uBPA measurements in spiked samples by four participating laboratories. Each graph (top to bottom) represents the data from an individual spiked sample ranging from the lowest concentration (0.5 ng/ml) to the highest concentration (19.5 ng/ml). B) Results reported for BPA-G measurements in spiked samples by four participating laboratories. Each graph (top to bottom) represents the data from an individual spiked sample ranging from the lowest concentration (0.5 ng/ml) to the highest concentration (19.5 ng/ml). In both panels, graphs represent mean ± standard deviations reported from each laboratory. The red line marks the actual concentration spiked and the yellow bar marks the range of ±20%. At the bottom of each panel is the performance summary for each laboratory for Phase 3 for uBPA (A) and BPA-G (B). A method was considered “verified” for the phase when at least 4 of 5 spiked samples measured concentrations within 20% of the actual spiked amount. Click here for file

Additional file 6: Figure S5 Concentrations of uBPA and BPA-G in human serum spiked with only BPA-G. BPA-G was reported by all four laboratories and low concentrations of uBPA were reported by two laboratories. Graph represents mean ± standard deviations reported from each laboratory. Click here for file
