# Erratum to: A round robin approach to the analysis of bisphenol a (BPA) in human blood samples

**DOI:** 10.1186/s12940-016-0126-z

**Published:** 2016-03-08

**Authors:** Laura N Vandenberg, Roy R Gerona, Kurunthachalam Kannan, Julia A Taylor, Richard B van Breemen, Carrie A Dickenson, Chunyang Liao, Yang Yuan, Retha R Newbold, Vasantha Padmanabhan, Frederick S vom Saal, Tracey J Woodruff

**Affiliations:** Division of Environmental Health Sciences, University of Massachusetts –Amherst, School of Public Health, Amherst, MA USA; Department of Laboratory Medicine, University of California – San Francisco, San Francisco, CA USA; Wadsworth Center, New York State Department of Health, and State University of New York at Albany, Albany, NY USA; Division of Biological Sciences, University of Missouri, Columbia, MO USA; College of Pharmacy, University of Illinois, Chicago, IL USA; Program on Reproductive Health and the Environment, Department of Obstetrics, Gynecology, and Reproductive Sciences, University of California – San Francisco, San Francisco, CA USA; Wadsworth Center, NY State Department of Public Health, Albany, NY USA; National Institute of Environmental Health Sciences, Research Triangle Park, NC USA; Department of Pediatrics and Reproductive Sciences Program, University of Michigan, Ann Arbor, MI USA

After publication of this work [1], we noted errors in grant funding acknowledged. NIEHS R01 HD31544 is an incorrect number and should be replaced with NIEHS R01 ES013527; NIEHS 1P01 ES02284401 contains a typo and should be changed to NIEHS P01 ES022841. Finally, USEPA P20 STAR RD83467801 and RD 83543601 are inconsistent with grant numbers listed in the U.S. Environmental Protection Agency’s database. The correct numbers are USEPA P20 STAR R834678 and USEPA R835433.

The corrected Acknowledgements have been included in full in this erratum.
