# Supplementary material for: A round robin approach to the analysis of bisphenol a (BPA) in human blood samples
Source: Environ Health. 2014 Apr 1;13:25. doi: 10.1186/1476-069X-13-25 (PMC4066311; doi:10.1186/1476-069X-13-25)
Supplement: Additional file 1: Table S1 — Liquid chromatography and mass spectrometry parameters used by Round Robin laboratories. [file 1476-069X-13-25-S1.doc]

# Additional file 1: Table S1: Liquid chromatography and mass spectrometry parameters used by Round Robin laboratories.

| **Method Parameter** | **Lab #1** | **Lab #2** | **Lab #3** | **Lab #4** |
| --- | --- | --- | --- | --- |
| Instrument (LC, MS) | Shimadzu LCMS-8080 | Agilent 1100 Series HPLC system, API 5500 electrospray triple quadrupole mass spectrometer | Agilent LC 1260- AB Sciex 5500 Triple Quad MS | LC: Thermo Scientific Accela HPLC. Mass spectrometer: Thermo Scientific TSQ Quantum Access MAX triple stage quadrupole |
| Column (type, brand, dimensions, particle size) | Shimadzu Shimpack XR-ODS III (2.0 mm x 50 mm, 1.6 µm) | C18, Betasil®(Thermo Electron Corporation) , 100 × 2.1 mm, 5 µm | Agilent Extend C-18 (4.6 x 100 mm, 1.8 μm) | C18 BDS, Hyperclone (Phenomenex), 100x4.6 mm, 3 micron |
| Column temperature | 50 °C | Room temperature | 50° C | Not heated. |
| Chromatographic elution (isocratic or gradient?)  If gradient, specify gradient profile | 1.5 min linear gradient | 15% methanol at 0 min, held for 2 min; increased to 75% methanol at 2 to 2.5 min, held for 2.5 min; then increased to 99% methanol at 5 to 10 min, held for 3.5 min; and finally reversed to 15% methanol, held for 6.5 min before the next injection. | Gradient  0-0.5 min= 30% MPB  0.5-1 min= 30-75% MPB  1-4 min= 75-100% MPB  4-6 min= 100% MPB  6.01-12 min= 30% MPB | Gradient. 0-2 min, 15% acetonitrile;  2-2.5 min, increased to 25% acetonitrile;  2.5-5 min, 25% acetonitrile;  5-9 min, increased to 97% acetonitrile;  9-14.5 min, 97% acetonitrile;  14.51-18 min, 15% acetonitrile. |
| Mobile phase(s) (composition, pH) | 10 to 100% acetonitrile with co-solvent of 1 mM aqueous ammonium acetate | methanol and 10 mM ammonium acetate (pH ~5) | Mobile Phase A (MPA): Water + 0.05% Ammonium acetate (pH 7.80)  Mobile Phase B (MPB): Methanol + 0.05% Ammonium acetate (pH 7.80) | A: 10:90 acetonitrile:H2O containing 0.01% ammonia (pH 9); B: Acetonitrile. |
| Flow rate | 400 µL /min | 300 µL/min | 500 µL /min | 300 µL /min |
| Injection volume | 5 µL | 10 µL | 25 μL | 10 µL |
| Chromatographic run length | 1.8 min | 20 min | 12 min | 18 min |
| Ion source/polarity | Electrospray/negative ion | Electrospray, negative | ESI negative | ESI, negative polarity |
| Mass transitions monitored | BPA  *m/z* 227 to *m/z* 212 (quantifier)  *m/z* 227 to *m/z* 133 (qualifier)  BPA-[d6]  *m/z* 233 to *m/z* 215  BPA-G  *m/z* 403 to *m/z* 227 (quantifier)  *m/z* 403 to 133 (qualifier)  BPA-G-[13C-12]  *m/z* 415 to 239 | 227>212 for BPA, 239>224 for ^13^C_12_-BPA and 403>113for BPA glucuronide | BPA  Quantifier: 227.0-133.1  Qualifier: 227.0- 212.1  BPA-d6  Quantifier: 233.0-85.0  Qualifier: 233.0- 122.8  BPA-G  Quantifier: 402.9-112.9  Qualifier: 402.9- 226.9  ^13^C_12_-BPA-G  Quantifier: 415.0-112.8  Qualifier: 415.0- 239.1 | BPA, m/z 227>212;  C13-BPA, m/z 239>224;  d6-BPA, m/z 233>138;  BPA-glucuronide, m/z 403> 227;  C13-BPA glucuronide, m/z 414> 239. |
