# Supplementary figures and images for: A round robin approach to the analysis of bisphenol a (BPA) in human blood samples
Source: Environ Health. 2014 Apr 1;13:25. doi: 10.1186/1476-069X-13-25 (PMC4066311; doi:10.1186/1476-069X-13-25)

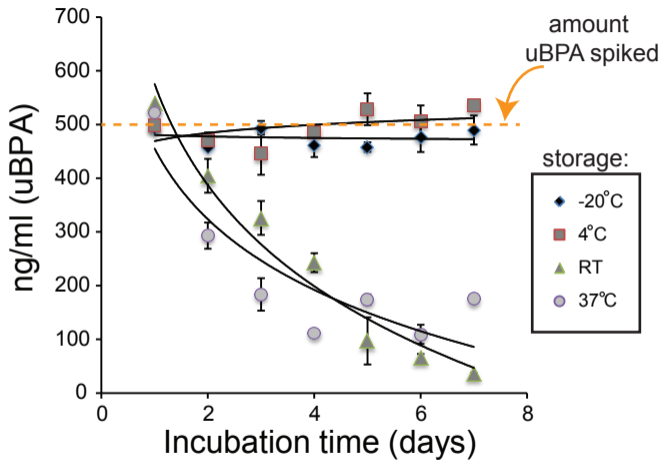

Supplement: Additional file 2: Figure S1 — Analyses of shipping conditions. Serum samples were spiked with 500 ng/ml uBPA and subjected to different storage conditions for up to 7 days. uBPA concentrations were stable when stored at -20°C or 4°C, but unstable at room temperature or 37°C. [file 1476-069X-13-25-S2.pdf]

A

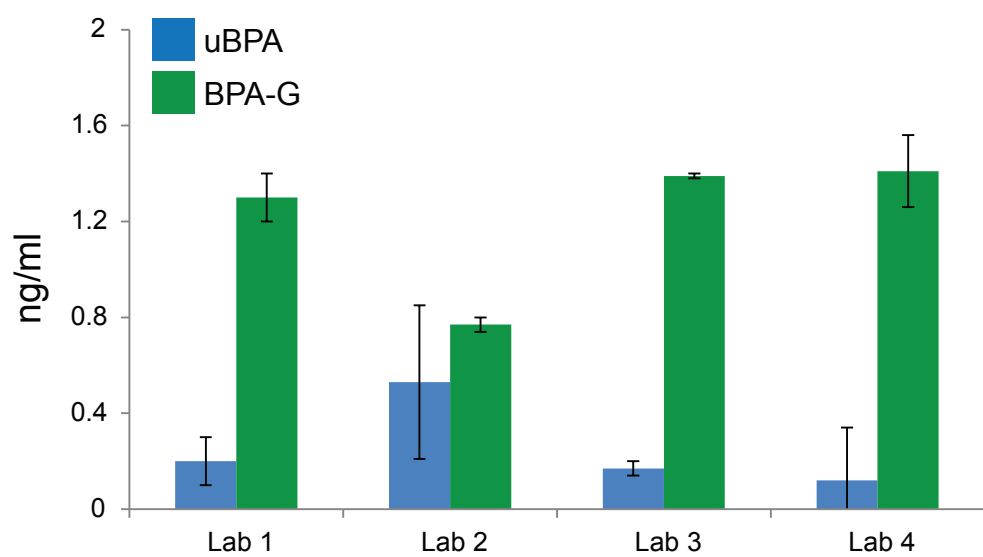

B

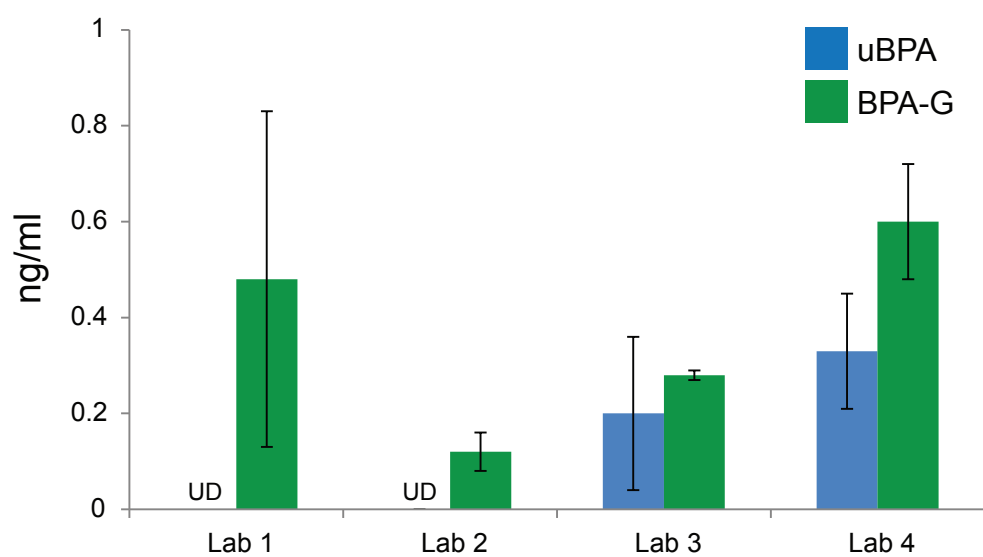

C

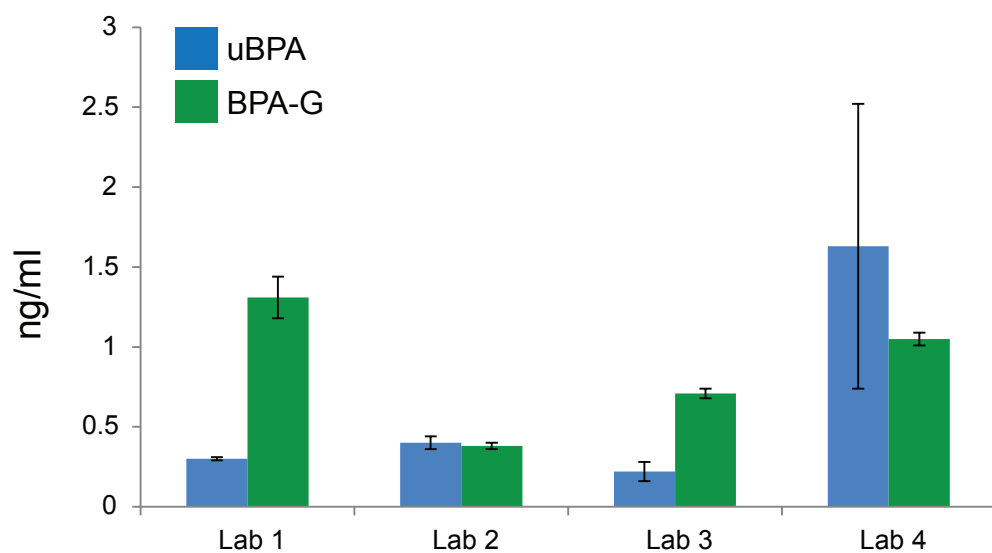

Supplement: Additional file 4: Figure S3 — uBPA and BPA-G were detected in unspiked pooled samples that were used for different Round Robin experiments. A) Concentrations of uBPA and BPA-G reported for the Phase 2 pooled samples that were used for spiked experiments with uBPA and BPA-G. B) Concentrations of uBPA and BPA-G reported for the Phase 3 pooled samples that were used for spiked experiments with uBPA and BPA-G. C) Concentrations of uBPA and BPA-G reported for the Phase 3 pooled samples that were spiked with BPA-G only. In all panels, graphs represent mean ± standard deviations reported from each laboratory. [file 1476-069X-13-25-S4.pdf]

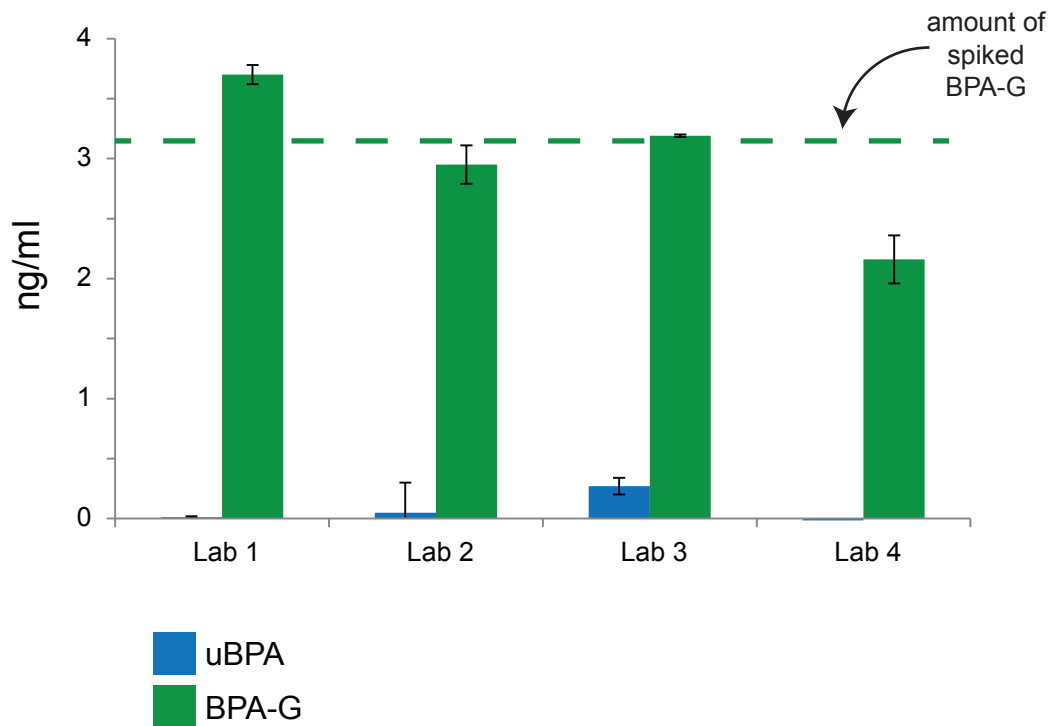

Supplement: Additional file 6: Figure S5 — Concentrations of uBPA and BPA-G in human serum spiked with only BPA-G. BPA-G was reported by all four laboratories and low concentrations of uBPA were reported by two laboratories. Graph represents mean ± standard deviations reported from each laboratory. [file 1476-069X-13-25-S6.pdf]
