# Supplementary material for: A round robin approach to the analysis of bisphenol a (BPA) in human blood samples
Source: Environ Health. 2014 Apr 1;13:25. doi: 10.1186/1476-069X-13-25 (PMC4066311; doi:10.1186/1476-069X-13-25)
Supplement: Additional file 3: Figure S2 — Linearity was observed by all laboratories for uBPA and BPA-G in spiked serum samples from Phase 3. A) Linear relationships were observed for uBPA in Phase 3 samples (spiked over the range of 0.5 to 19.53 ng/ml) by all four laboratories. B) When analyses were limited to only the three samples spiked with the lowest concentrations of uBPA (0.5 to 3.13 ng/ml), laboratories were still able to distinguish low, moderate and high concentrations of uBPA. C) Linear relationships were also observed for BPA-G in all Phase 2 samples (spiked over the range of 0.5 to 19.53 ng/ml) by all four laboratories. D) When analyses were limited to only the three samples spiked with the lowest concentrations of BPA-G (0.5 to 3.13 ng/ml), all laboratories were still able to distinguish low, moderate and high concentrations. [file 1476-069X-13-25-S3.pdf]

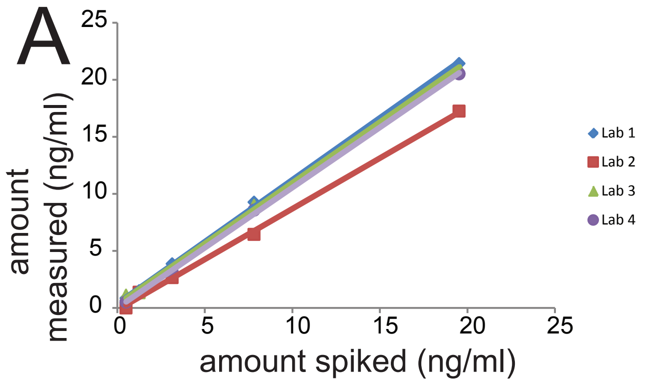

	slope	intercept	R square
Lab 1	1.08	0.4	0.9991
Lab 2	0.89	-0.17	0.9981
Lab 3	1.06	0.33	0.9988
Lab 4	1.05	0.02	0.9995

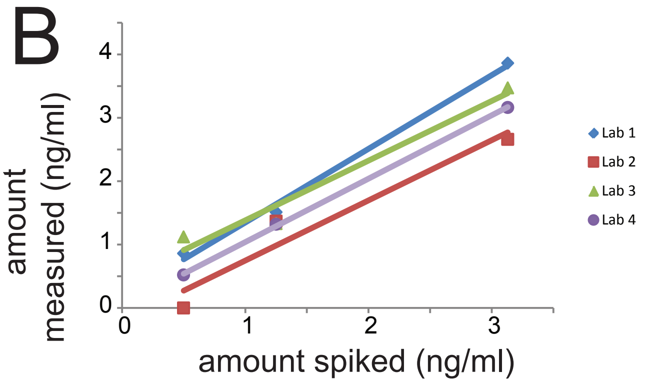

	slope	intercept	R square
Lab 1	1.16	0.19	0.9947
Lab 2	0.95	-0.2	0.9337
Lab 3	0.94	0.44	0.9607
Lab 4	1	0.04	0.9996

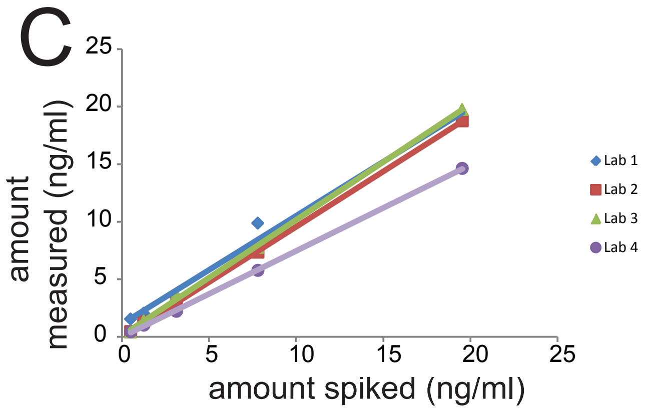

	slope	intercept	R square
Lab 1	0.94	1.12	0.9877
Lab 2	0.96	-0.04	0.9998
Lab 3	1	0.12	0.999
Lab 4	0.75	-0.01	0.9998

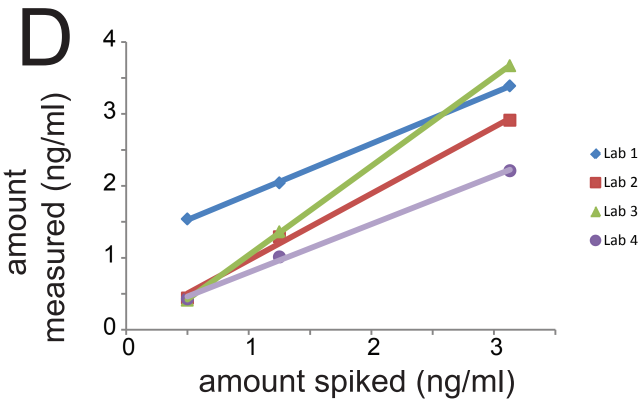

	slope	intercept	R square
Lab 1	0.71	1.17	0.9997
Lab 2	0.92	0.04	0.9958
Lab 3	1.24	-0.2	1
Lab 4	0.67	0.13	0.998
