# Supplementary material for: A round robin approach to the analysis of bisphenol a (BPA) in human blood samples
Source: Environ Health. 2014 Apr 1;13:25. doi: 10.1186/1476-069X-13-25 (PMC4066311; doi:10.1186/1476-069X-13-25)
Supplement: Additional file 5: Figure S4 — Accuracy of spiked samples, Phase 3. A) Results reported for uBPA measurements in spiked samples by four participating laboratories. Each graph (top to bottom) represents the data from an individual spiked sample ranging from the lowest concentration (0.5 ng/ml) to the highest concentration (19.5 ng/ml). B) Results reported for BPA-G measurements in spiked samples by four participating laboratories. Each graph (top to bottom) represents the data from an individual spiked sample ranging from the lowest concentration (0.5 ng/ml) to the highest concentration (19.5 ng/ml). In both panels, graphs represent mean ± standard deviations reported from each laboratory. The red line marks the actual concentration spiked and the yellow bar marks the range of ±20%. At the bottom of each panel is the performance summary for each laboratory for Phase 3 for uBPA (A) and BPA-G (B). A method was considered “verified” for the phase when at least 4 of 5 spiked samples measured concentrations within 20% of the actual spiked amount. [file 1476-069X-13-25-S5.pdf]

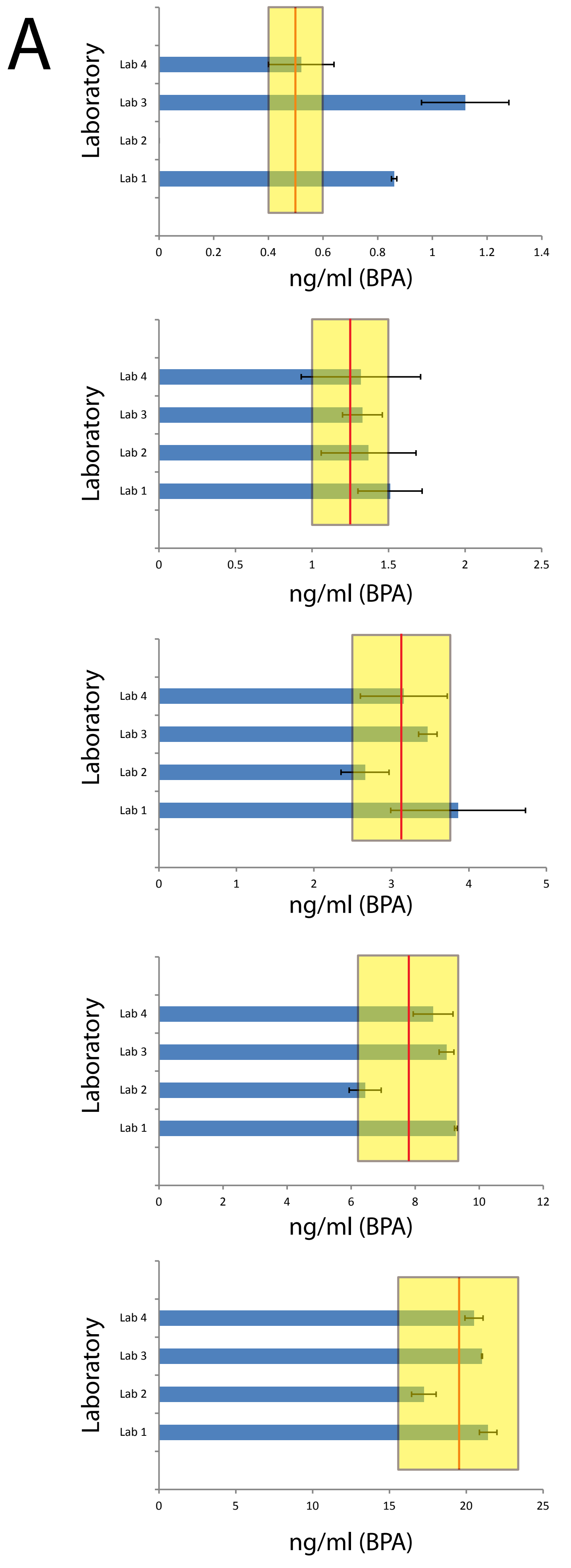

spiked:	0.5	1.25	3.13	7.81	19.53	result
Lab 1	no	no	no	PASS	PASS	
Lab 2	no	PASS	PASS	PASS	PASS	VERIFIED
Lab 3	no	PASS	PASS	PASS	PASS	VERIFIED
Lab 4	PASS	PASS	PASS	PASS	PASS	VERIFIED

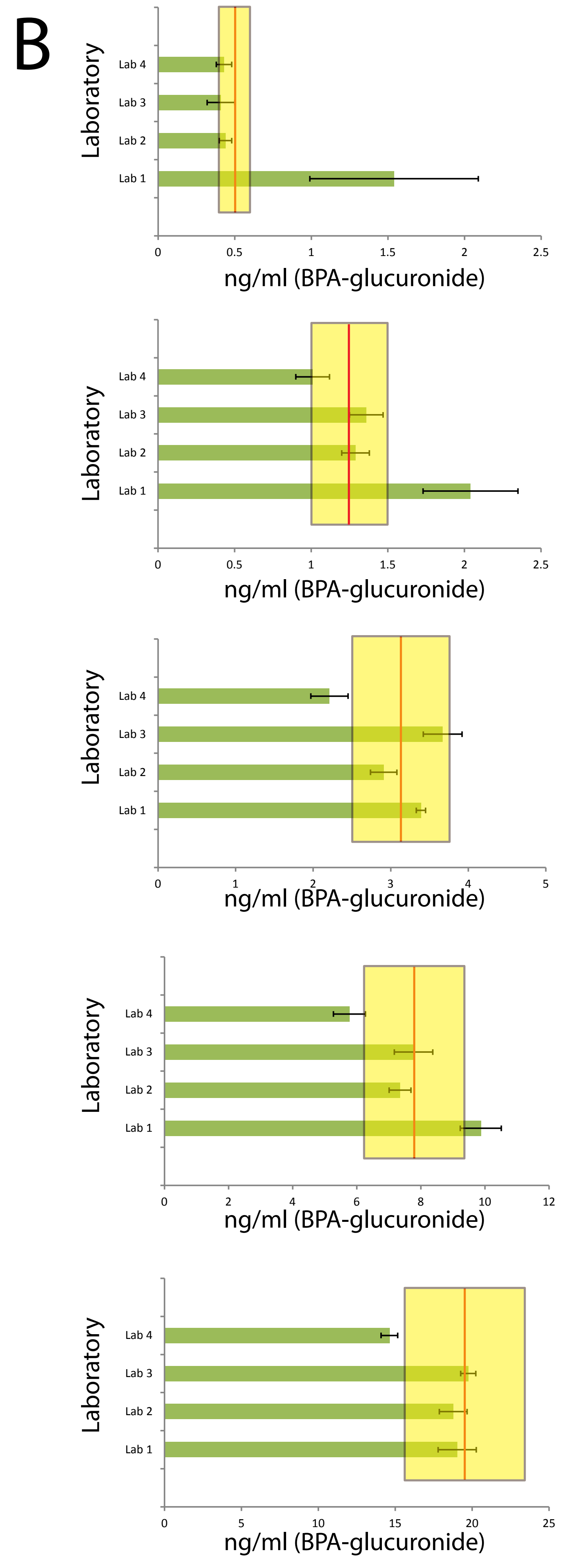

spiked:	0.5	1.25	3.13	7.81	19.53	result
Lab 1	no	no	PASS	no	PASS	
Lab 2	PASS	PASS	PASS	PASS	PASS	VERIFIED
Lab 3	PASS	PASS	PASS	PASS	PASS	VERIFIED
Lab 4	PASS	PASS	no	no	no	
